# A comparison of the efficacy and safety of complementary and alternative therapies for premature ovarian insufficiency: A protocol for network meta-analysis

**DOI:** 10.1097/MD.0000000000021538

**Published:** 2020-07-31

**Authors:** Lei Zhang, Honglin Li, Jianwei Zhang, Xinliang Kong, Zhijuan Wu, Shuangqian Dong, Xiuyun Qin

**Affiliations:** a The First Clinical College, Shandong University of Traditional Chinese Medicine; b Hospital Affiliated to Shandong University of Traditional Chinese Medicine; c The College of Traditional Chinese Medicine, Shandong University of Traditional Chinese Medicine, Jinan; d Rizhao Maternal and Child Health Care Hospital, Rizhao, Shandong Province, China.

**Keywords:** complementary and alternative therapies, network meta-analysis, premature ovarian insufficiency, protocol

## Abstract

**Background:**

With the increase in the incidence of premature ovarian insufficiency (POI) over the years, the ovarian function has become one of the integral aspects of research in reproductive medicine today. POI seriously affects the physical and mental health of women, especially reproductive health. Studies show both complementary and alternative therapies to be effective in treating POIs. However, consistency in conclusions is still far-fetched. In light of this, we will carry out a study to evaluate the effectiveness and safety of complementary and alternative therapies for POIs. We therefore develop a study protocol for a proposed network meta-analysis (NMA) and systematic review on POI.

**Methods:**

The following electronic bibliographic database will be searched: VIP database, Wanfang database, Chinese National Knowledge Infrastructure (CNKI), The Cochrane Library, PubMed, EMBASE and Web of Science from inception till 31 December 2019. A search at the World Health Organization (WHO) International Clinical Trials Registry Platform will also be done. Subsequently, the searched data will undergo independent screening, retrieving, and risk of bias assessment by 2 reviewers. Analysis will be performed on included studies using the NMA technique. Next, the primary outcomes will be compared using ADDIS 1.16.5 and Stata 15.0.

**Results:**

The safety and effectiveness of alternative and complementary therapies used in the treatment of POI will be compared and evaluated.

**Conclusion:**

This work will provide high-quality evidence for clinicians in the field to build on for best practices in effective interventions (complementary and alternative therapies) for POI.

**Ethics and dissemination:**

This NMA is a secondary research which based on some previously published data. Therefore, the ethical approval was not necessary.

**PROSPERO registration number:**

CRD42020163873

## 1 Introduction

Premature Ovarian Insufficiency (POI) is a series of clinical syndromes (elevated gonadotropins and low estradiol levels) caused by ovarian dysfunction (less than 40 years of age), resulting in an altered or disturbed menstrual period (amenorrhea or oligomenorrhea).^[1,2]^ A decline in women's quality of life is also directly related to the disease. POI brings severe psychological pressures on women in reproductive age as well as long-term sequelae such as menopausal symptoms (hot flushes, night sweats, sexual dysfunctions, and insomnia), bone loss, low energy, impaired memory, labile mood, and related heart diseases.^[3]^ Therefore, early diagnosis, integrated, and individualized patient treatment plans are of utmost importance to a woman's quality of life, especially in childbearing age.

Unfortunately, it is a complicated etiology disease and lack of effective clinical treatment. POIs are usually associated with autoimmunity, iatrogenicity, genetics, environmental factors, and poor lifestyle.^[4]^ It has been determined that at least 20% to 25% of women with POI have a genetic basis, including chromosomal abnormalities and gene mutation.^[5–7]^ In China, however, the frequency of pathogenic gene mutations in POI patients is generally <2%, although clinical diagnostic reports concerning these values are limited.^[8]^ Common iatrogenic factors include surgery, radiotherapy, and chemotherapy.^[9]^ Besides, Some POI patients have autoimmune diseases, and it is most related to autoimmune thyroid disease.^[10]^

POI, generally affects approximately 1% of women before the age of 40, although its prevalence remains uncertain.^[11]^ Hormone replacement therapy (HRT), if there are no contraindications, however, should be given all POI patients, as HRT not only relieves symptoms of low estrogen but also prevents cardiovascular diseases and osteoporosis.^[1,12,13]^ The use of HRT may, however, increase the risk of venous thromboembolism, although, depending on the dose and timing of initiation.^[14]^ In developed countries, there is a growing public interest in and use of therapies outside the traditional (customary) western medical practice.^[15]^ In the UK and other European countries as well as Australia, complementary and alternative medicines are now mainstream, as the use of herbal medicines is widely accepted.^[16–19]^

Presently, women with POI also seek out complementary and alternative therapies, such as excise, acupuncture, Chinese herbal medicine, acupressure, breathing, hypnotherapy, massage, and meditation. Zhang and colleagues through a randomized controlled trial and found that “Tiaoren Tongdu acupuncture” is beneficial to POI of kidney deficiency.^[20]^ Also, treatments using Chinese herbal formulas carried out by a group of Chinese researchers proved useful in improving symptoms and regulating hormonal levels as compared to placebo in women with POI.^[21,22]^ However, most alternative and complementary therapies lack safety data and limited sufficient evidence.^[23]^ Notwithstanding, nonhormonal alternatives such as lifestyle changes, psychosomatic techniques, diet management and supplementation, prescription therapy, and other strategies are, however, still available and useful.^[24]^ These are nonhormonal alternatives. Many complementary and alternative therapies lack sufficient, high-quality evidence to support their prevention and treatment under different conditions.

The objectives of this study are to assess the conventional applications of alternative and complementary treatments available for POI patients and to summarize the potential benefits. A network meta-analysis (NMA) was applied in this research although similar studies were not available.

## 2 Materials and methods

### 2.1 Study registration

The protocol is conducted based on the Preferred Reporting Items for Systematic Review and Meta-Analysis Protocols (PRISMA-P) guidelines to conduct this study. This NMA has been registered in the International Prospective Systematic Registration Review (PROSPERO) and the registration number is CRD42020163873.

### 2.2 Inclusion criteria

All relevant complementary and alternative randomized controlled trials (therapies) for POI were factored in.

#### 2.2.1 Types of patients

The type of patients was selected on the basis of the European Society of Human Reproduction and Embryology (ESHRE) POI diagnostic criteria,^[1]^ which included vthe following:

1. Age less than 40 years;
2. oligo/amenorrhea for at least 4 months;
3. an elevated FSH level 25 IU/l on 2 occasions 4 weeks apart.

#### 2.2.2 Interventions

The treatment group will receive alternative and complementary therapies, such as moxibustion, acupuncture, Chinese herbal drugs, and topical heat. Besides, combined interventions with other treatments will be included. The control group will include placebo, no treatment, sham acupuncture, HRT, and western medicine.

#### 2.2.3 Outcomes

The main outcome indicators are:

1. Clinical total effective rate;
2. Decrease rate of symptom integral;
3. Improvement of menstrual symptoms;
4. Comparison of serum FSH, LH, and E2 levels between day 2 to day 5 of the menstrual cycle.

Secondary outcome indicators are

1. Anti Mullerian Hormone (AMH) levels;
2. Antral follicle count between day 2 to day 5 of the menstrual cycle (assessed by transvaginal ultrasound);
3. Endometrial thickness;
4. Adverse events.

#### 2.2.4 Exclusion criteria

Patients who do not conform to the above diagnostic criteria or are without diagnostic criteria.

### 2.3 Search strategy

The following electronic bibliographic database were searched: VIP database, Web of Science, Wanfang database, Chinese National Knowledge Infrastructure (CNKI), PubMed, The Cochrane Library, and EMBASE since inception till 31 December 2019. Searches in World Health Organization (WHO) International Clinical Trials Registry Platform will also be done. The MeSH terms and free words will be used to construct the retrieval formula. The selection will not be limited to the publication status, year of publication, country or date. Taken as example, the following (Table 1) is the specific search process for PubMed.

**Table 1:**
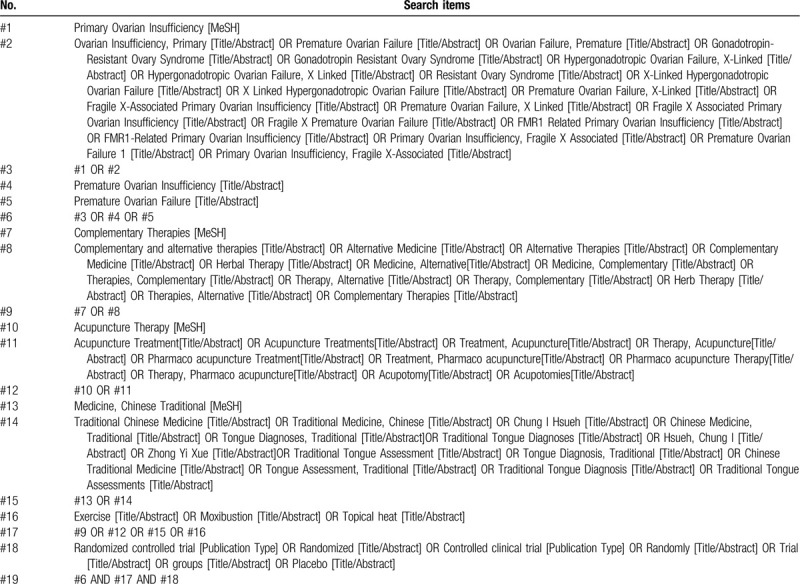
Search strategy for the PubMed.

### 2.4 Study selection and data collection

#### 2.4.1 Note Express 3.2.0 software will be used for document management

Firstly, duplicate pieces of literature will be checked by software and manually. Then, abstracts and keywords will be read to eliminate inconsistent literature. Finally, after eliminating the inconsistent literature, what is available will be downloaded and reevaluated.

#### 2.4.2 Data collection

Two independent researchers will extract the literature and establish a database of Excel, which will mainly include: the title, author, publish time, number of patients, the patients’ essential characteristics, ways of intervention, observe outcome indicators, and results. A third researcher would be invited to discuss and resolve disagreements were differences in opinion exists.

### 2.5 Literature quality evaluation

Two independent reviewers will assess risk of bias among studies with Cochrane Collaboration's tool. The following 7 domain categories will be applied in this exercise: blinding of personnel and participants (performance bias); and other bias; blinding of outcome assessment (detection bias); incomplete outcome data (attrition bias); selective outcome reporting (reporting bias); random sequence generation (selection bias); allocation concealment (selection bias).^[25]^ In case of disagreements, a third reviewer will called upon.

### 2.6 Statistical analysis

First, the network evidence graph was drawn and consistently tested by Stata15.0 software. Then, direct comparisons’ meta-analysis will be performed by Rev Man5.3 software, and NMA will be performed by ADDIS 1.16.8 software. Odds ratios and 95% credibility intervals (CrI) will be used to be reported, and a ranking table will be conducted for each intervention. *P* value <.05 and 95%CrI will be used as the criteria for statistical differences. ADDIS software mainly uses the potential scale reduction factor to evaluate the convergence of the results. It indicates that the results have good convergence performance and the analysis is reliable when potential scale reduction factor is close to or equal to 1.

### 2.7 Subgroup analysis

If *I*^2^ > 50%, heterogeneity sources will be determined through a subgroup analysis. A detailed subgroup analysis will be listed.

1. Patient characteristics: age and course of the disease.
2. Interventions: acupuncture; traditional Chinese medicine; psychosomatic techniques; exercise and other treatments.

### 2.8 Sensitivity analysis

As a commonly used method, sensitivity analysis will be applied to check the certainty of results and evaluate the effect of each study with a high risk of bias.

### 2.9 Grading the quality of evidence

GRADE Pro V3 software will be utilized to check the quality of the GRADE handbook evidence. The evaluation process will be assessed from the standpoints of limitations, publication bias inconsistency, imprecision, and indirectness. The results of the evidence would then be categorized in four quality grades: shallow, low, moderate, and high quality.

## 3 Discussion

Recently, as the number of people with POI increases in China, these group of women now tend to seek complementary and alternative therapies. To the best of our knowledge, although there are many researches on the effectiveness of traditional Chinese medicine and acupuncture in POI management, the evaluation and comparison of various treatment methods are insufficient. This study aims to provide more convincing and detailed information on complementary and alternative therapies to improve symptoms of POI. We searched databases for systematic reviews and NMA; however, data were lacking. NMA will demonstrate an effective treatment for POI, providing clinicians with evidence-based resources and the confidence to use them.

Some restrictions may affect the conclusions drawn in this research protocol. Though there are several complementary and alternative therapies, however, the subgroup analysis set up in this program were mainly Chinese medicine, acupuncture, exercise therapy, psychosomatic techniques, and other therapies. They may be heterogeneous. Finally, due to the language barrier of the researchers, they decided to consult only English and Chinese literature, which may, however, have potentially lead to a few missing essential pieces of literature.

## Author contributions

**Conceptualization:** Lei Zhang, Jianwei Zhang.

**Data curation:** Lei Zhang, Honglin Li, Xinliang Kong, Jianwei Zhang.

**Methodology:** Lei Zhang, Jianwei Zhang, Honglin Li, Zhijuan Wu.

**Project administration:** Zhijuan Wu.

**Search strategy:** Lei Zhang, Xinliang Kong, Zhijuan Wu, Jianwei Zhang.

**Software:** Lei Zhang, Jianwei Zhang, Honglin Li, Xinliang Kong, Zhijuan Wu.

**Statistical analysis:** Lei Zhang, Honglin Li, Xinliang Kong.

**Writing – original draft:** Lei Zhang.

**Writing – review & editing:** Lei Zhang.
